# Fuel-Powered Soft Actuators: Emerging Strategies for Autonomous and Miniaturized Robots

**DOI:** 10.1007/s40820-025-01969-w

**Published:** 2026-01-04

**Authors:** Cheng Zhou, Zhoutao Li, Hailong Wei, Guorong Zhang, Fengrui Zhang, Xiaoshuang Zhou, Hongwei Hu, Guanggui Cheng, Jianning Ding, Shi Hyeong Kim, Ray H. Baughman, Xinghao Hu

**Affiliations:** 1 School of Mechanical Engineering, Jiangsu University, Zhenjiang, 212013 People’s Republic of China; 2 School of Microelectronics and Control Engineering, Changzhou University, Changzhou, 213164 People’s Republic of China; 3 Department of Advanced Textile R&D, Korea Institute of Industrial Technology, Ansan-Si, Gyeonggi-do 15588 Republic of Korea; 4 Department of Advanced Materials Engineering, Chung-Ang University, Anseong, 17546 Republic of Korea; 5 Alan G. MacDiarmid NanoTech Institute, University of Texas at Dallas, Richardson, TX 75080 USA

**Keywords:** Fuel-powered soft actuators, Fuel electrochemical actuators, Fuel thermal actuators, Fuel-pneumatic actuators

## Abstract

Soft actuators, capable of producing mechanical work in response to external stimuli, have potential applications in robotics and exoskeletons. However, they face major challenges related to energy supply, especially in long-distance and miniaturized environments. Fuel-driven actuators offer a promising solution by enabling the conversion of chemical energy into mechanical energy, supporting self-sustaining operations. Chemical energy from fuel can be converted into mechanical energy either directly or indirectly through methods such as electron transfer-induced charge injection, structural changes, fuel-to-electricity conversion, fuel combustion-induced heat, or fuel-induced pneumatic actuation. This paper provides a comprehensive review of recent developments in fuel-powered actuators, covering their fundamental principles, advancements, and challenges. It concludes with an outlook for miniaturized and autonomous robots, highlighting the great potential of integrating fuel-powered actuators.

## Highlights

- Fuel-powered soft actuators are elucidated in terms of their high power densities, enabling robots to operate effectively in long-distance or miniaturized environments.
- The working principles, applications, and potential future improvements of typical fuel-powered actuators are comprehensively reviewed and discussed
- Existing challenges and the future pathways for fuel-powered soft robots are delineated.

## Graphical abstract

**Figure:**
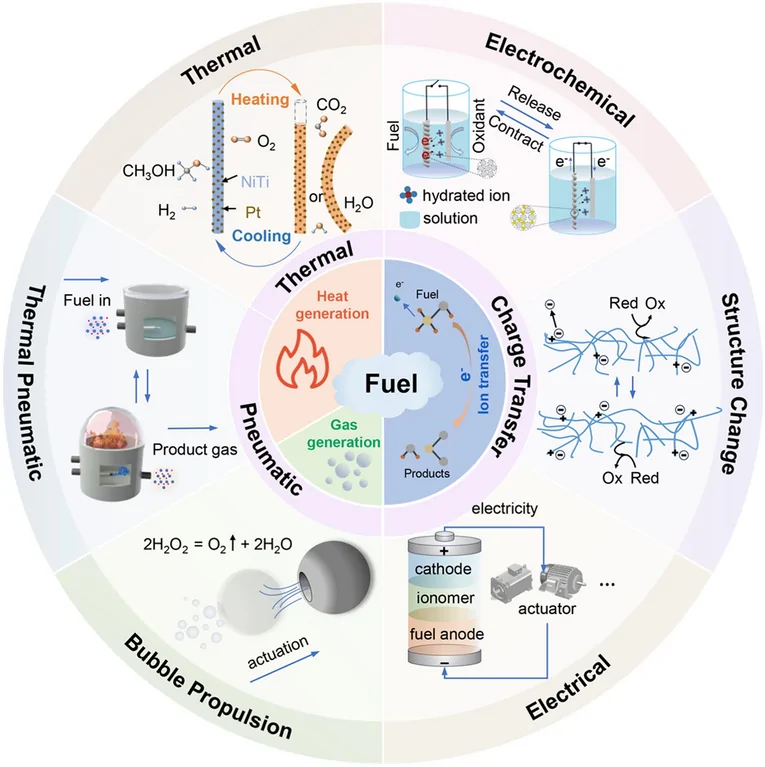

## Introduction

Soft actuators have gained widespread interest in the fields of medical robotics (e.g., minimally invasive surgical tools [1–7]) and intelligent bionic systems (e.g., flapping-wing aircraft [8–10]) because of their flexibility, safety, and silent operation. Generally, environmentally powered actuators such as solvent-driven yarn muscles [11, 12] and light-driven bending actuators [13–15] operate well, but they rely on the environmental conditions. Hydraulic or electrical stimulation is commonly used as a driving method since these methods provide fast response speeds and are easy to control [16–20]. However, these actuators are often tethered or require large batteries, which restricts their development for autonomous, compact, or long-distance robot applications. Fuel has a higher energy density than lithium-ion batteries, making it an excellent power source for robots. Using fuel to power soft actuators presents an effective solution for applications that need sustained, continuous movement, such as space exploration and ocean exploration.

Typically, when fuel reacts with a catalyst, the electrons of the fuel transfer to the catalyst or the oxidant. Once they are immersed in electrolytes, these transferred electrons and the associated generated electricity can be used to power robots [21–25]. Inspired by biological muscles, electroactive polymers (EAPs) and carbon nanotubes (CNTs) have been developed to emulate the energy transfer mechanisms of adenosine triphosphate (ATP) [26–29] by changing their shape in response to a redox potential. These actuators directly transfer the chemical energy of fuel into mechanical energy.

In addition, chemical reactions of fuel are often accompanied by heat or gas, as seen in the combustion of hydrogen or methane [30, 31], which can also be harnessed for actuation. This approach enables an indirect conversion of chemical energy into mechanical energy via thermal (called fuel-driven thermally actuators) or pneumatic energy (called fuel-driven pneumatically actuators). Fuel-driven thermally actuators, such as shape memory alloys (SMA) [32], utilize thermal energy generated from fuel reaction to actuate, eliminating reliance on external batteries. Similar to traditional pneumatic actuators [33], the pressure produced by these fuel reactions can drive actuation. For instance, the decomposition of hydrogen peroxide stored within a robot’s body creates high-pressure gas to facilitate movement [34, 35]. Advances in fuel-powered actuation technologies have been rapidly developed in recent years, leading to the emergence of various autonomous robots and self-powered actuators. There are a few reviews on fuel-powered actuators in the literature. These reviews focus on ion-induced structure changes of conductive polymers [36] and the gas evolution reactions (GERs) for pneumatic actuators [37, 38]. There is currently no literature that summarizes the latest developments in the operational principles of fuel-powered autonomous, miniaturized, and long-duration actuators.

**Fig. 1:**
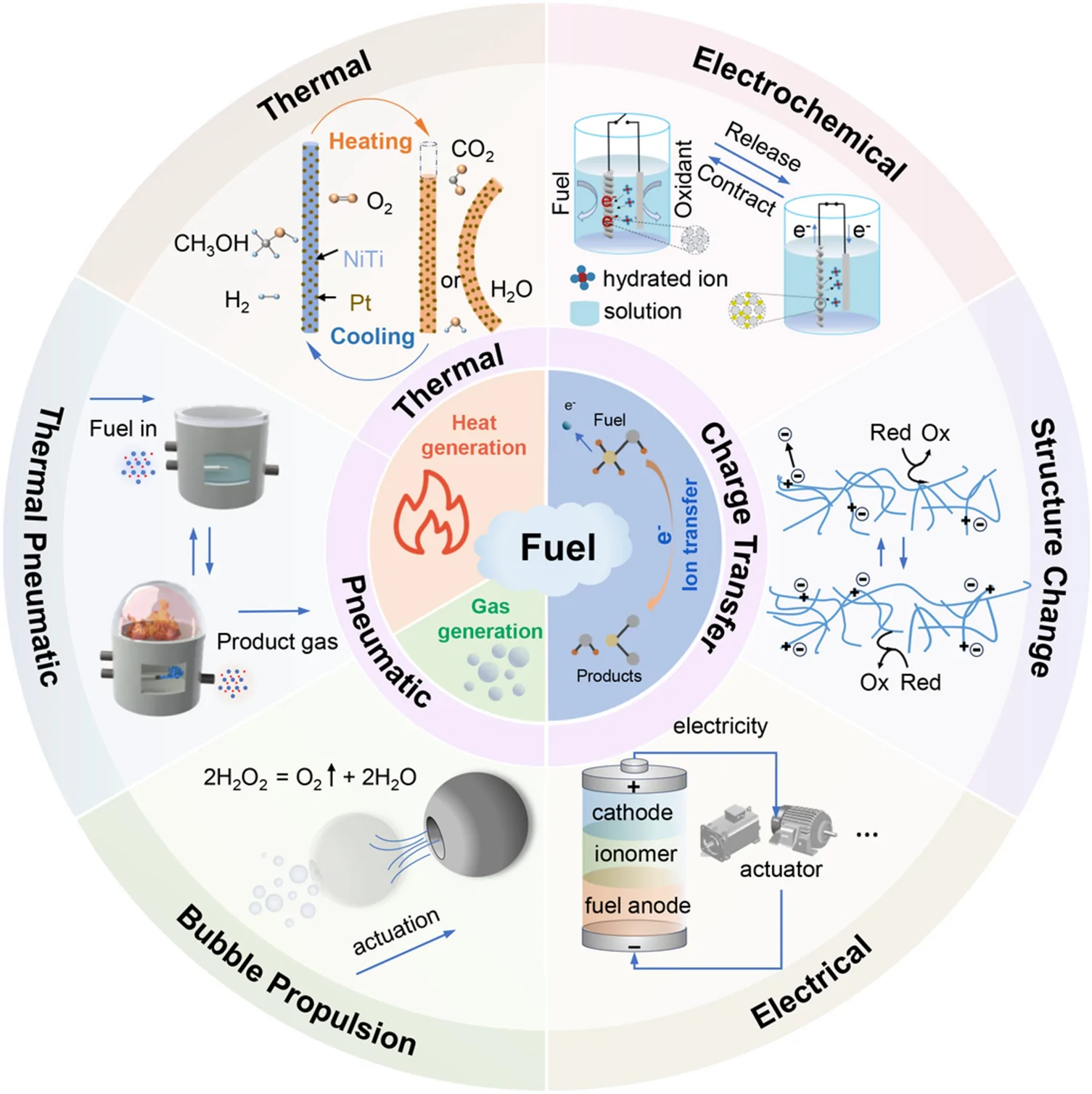
Overview of various fuel-powered soft actuators

This paper comprehensively reviews the latest advancements in various actuation technologies for autonomous and miniaturized robots that utilize fuel sources (Fig. 1). The conversion of chemical energy from fuels into mechanical energy can be achieved through various approaches, including electron transfer-induced charge injection or polymer structural changes, fuel combustion-induced thermal actuation, and fuel-induced pneumatic actuation. We thoroughly summarize the working principles, typical examples, applications, and potential future improvements of these actuators. Through a review of the literature, we highlight the importance of chemical fuels in powering autonomous and miniaturized robots, as well as the challenges these technologies may face.

## Fuel-Powered Artificial Muscles Using Charge Transfer-Induced Actuation

**Fig. 2:**
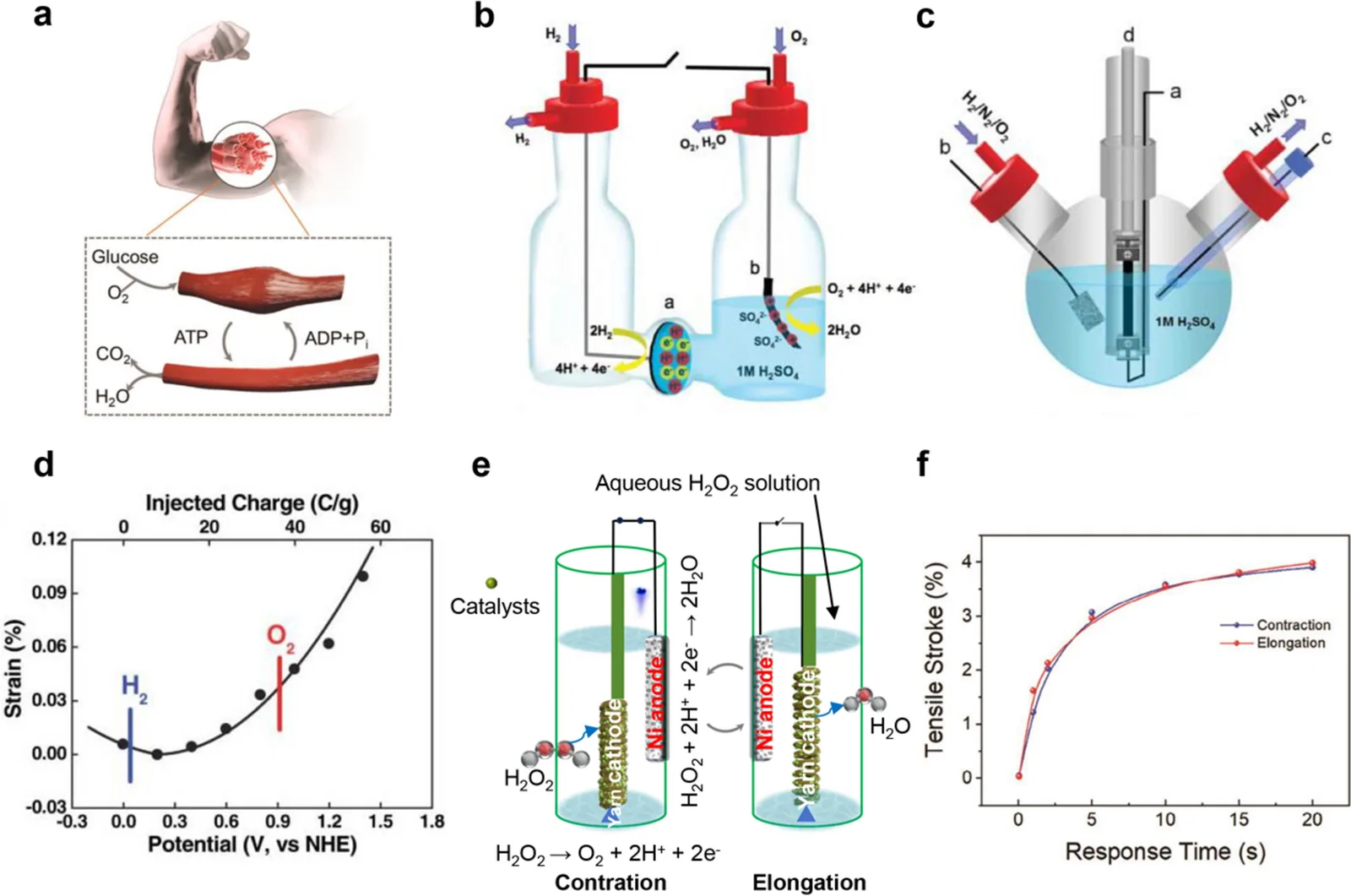
Fuel-powered yarn artificial muscles driven by fuel reaction-induced ion injection. **a** Schematic diagram of the working principle of human skeletal muscles [58]. **b** Schematic diagram of a cantilever-based two-compartment fuel cell CNT film muscle [54]. **c** Schematic diagram of a one-compartment CNT film muscle [54]. **d** The measured hydrogen and oxygen potentials for the fuel-powered CNT film actuator [54]. **e** Schematic illustration of the working process of one-compartment coiled CNT yarn artificial muscle [58]. **f** The tensile stroke as a function of time during yarn muscle contraction and elongation [58]

In nature, biological skeletal muscles use ATP to achieve self-powered, autonomous, and efficient actuation. Upon receiving nerve signals, ATP is converted to adenosine diphosphate (ADP), which drives conformational changes in nanoscale proteins (Fig. 2a). This process facilitates sliding of actin filaments, ultimately resulting in muscle contraction [39–41]. Inspired by skeletal muscles, the electrochemical actuators and electroactive polymers (EAPs) were developed. The electrochemical actuators involve the injection of charges into the electrochemical double layer of the actuator [42] and incorporate various phenomena such as pseudo-capacitance [43, 44], redox reactions [26, 45, 46], and ion de-/intercalation [47]. The movement of these charges results in volumetric changes within the actuator, causing it to either expand or contract.

To avoid the use of external electrochemical power, fuel-driven chemical muscles were conceived. These muscles function as fuel cell electrodes to generate potential. They catalyze the fuel, resulting in the generation and capacitive storage of electrical energy, while also causing actuation when the inter-electrode circuit is open. In this section, we summarize the advancements in fuel-electrochemical artificial muscles.

### Two-Compartment Fuel-Powered Artificial Muscles

Hydrocarbon-based fuel cells are widely studied for generating large electric power since they can deliver energy densities that are approximately 2–50 times greater than those of lithium-ion batteries [48–50]. Carbon nanotube (CNT) yarns possess a large electrochemical surface area, making them an attractive option for use in electrochemically driven artificial muscles [51–53]. Baughman's group first reported a fuel-powered CNT film artificial muscle that converts the chemical energy from high-energy density fuels (H_2_) into mechanical energy [54]. As shown in Fig. 2b, the muscle system comprises a hydrogen-oxidation membrane electrode (2H_2_ → 4H^+^ + 4e^−^, counter electrode) and an oxygen-reduction electrode (O_2_+ 4H^+^+ 4e^−^→ 2H_2_O, actuating electrode). These two electrodes are mechanically uncoupled but ionically connected through a 1 M aqueous H_2_SO_4_ solution to realize actuation during charge and discharge. The fuel cell muscle that uses two separate electrodes is called a two-compartment cell muscle.

This fuel cell muscle utilizes a catalyst-containing CNT sheet electrode to function as the actuating electrode. When the inter-electrode circuit is open, the Pt reduces oxygen gas at the sheet electrode, and four electrons are extracted from the CNT sheet electrode. The potential of the sheet electrode starts to increase. Simultaneously, the sheet electrode absorbs the solvated SO_4_^−^ ions to serve as countercharges, thereby causing actuation. As the potential increased to 0.8 V, a 3-cm CNT strip can deflect 2 mm within 5 s. Upon short-circuiting the two electrodes, electrons from the counter electrode are injected into the CNT sheet, resulting in the desorption of solvated ions, causing the opposite actuator deflection. This fuel-powered chemical muscle combines energy conversion and actuation capabilities, producing electrical potential in open-circuit conditions and completing reciprocating mechanical motion when powered. It directly converts the chemical energy of the fuel into mechanical energy without any heat dissipation.

Two-compartment fuel cell muscles offer numerous advantages: They improve reaction efficiency by isolating the anode and cathode reaction processes, which effectively reduces side reactions. This configuration also simplifies reactant management. Moreover, these systems are known for their low heat loss and exceptional energy conversion efficiency. Notably, to maintain effective separation of the fuel within the two compartments, a sophisticated sealing part is essential between the chambers.

### One-Compartment Fuel-Powered Artificial Muscles

The two-compartment cell requires ion exchange membranes to maintain the separation of fuel and oxidant within their respective compartments, which adds to the structural complexity. To address this issue, a one-compartment fuel cell muscle has been developed, eliminating the necessity for ion exchange membranes. The first reported one-compartment fuel muscle is the CNT sheet muscle, which uses hydrogen and oxygen as the fuel source. Specifically, the actuating CNT sheet muscle serves as both reduction and oxidation electrodes, alternatively filling the cell with hydrogen and oxygen with an N_2_ purge in between (Fig. 2c) [54]. The platinum catalyst within the CNT sheet can catalyze both hydrogen and oxygen. The potential of the sheet is 0 V when filled with H_2_, and it increases to ~ 0.9 V when filled with O_2_ (Fig. 2d). This change in potential causes charges in the electrolyte to be injected into the sheet, leading to muscle contraction. Switching the external fuel gas with oxygen makes a reversible actuation. Note that the time to dissolve fuel gas or oxygen into the electrolyte slows the actuation speed. Nonetheless, the overall actuation stroke is the same as that achieved by using externally applied internal-electrode voltage. Similar to the case of electrically powered actuation using double-layer charge injection, the amount of charge injected into the two electrodes depends only on their electrochemical capacitance. However, the muscle stroke is quite small since the sheet muscle cannot generate a large stroke.

Twisted and coiled (TCP) yarn muscles use stimuli-induced yarn volume expansion to facilitate muscle untwisting, which in turn enables tensile actuation in TCP muscles [17, 55–57]. Inspired by these TCP muscles, Zhou et al. introduced a one-compartment, fuel-powered yarn muscle that uses a coiled CNT yarn as the actuating electrode [58]. As shown in Fig. 2e, hydrogen peroxide (H_2_O_2_) serves as the fuel, dissolved in an aqueous electrolyte comprising 0.15 M NaCl and 0.1 M HNO_3_. The decomposition of H_2_O_2_ occurs through a disproportionation reaction, eliminating the need for an additional oxidant. The system incorporates two electrodes: one is embedded with the reduction catalyst, Fe_3_[Co(CN)_6_]_2,_ within the coiled CNT yarn. The other, composed of nickel foam, acts as the oxidation catalyst and the anode. As a result, this CNT muscle achieves a reversible contractile stroke of 4% in just 20 s (Fig. 2f). The one-compartment design prioritizes operational safety, owing to mild reaction conditions and a reduced likelihood of generating explosive gas. Nevertheless, the presence of cobalt cyanide in the catalyst for H_2_O_2_ decomposition raises potential health risks for biological organisms.

### Polymer Structure Change Induced by Redox Reaction

The ions generated from fuel reactions can induce changes in yarn volume not only through injection but also by causing structural deformations within the material. Conductive polymers (CP) represent a category of volume change materials in which these volume changes arise from the reversible reduction and oxidation of the polymer, operating at low voltages of 0.1 ~ 3.0 V [59, 60]. The volume change is driven by the electrochemically induced insertion and desorption of ions, which results in conformational changes in the polymer chains [46]. Importantly, the reduction or oxidation processes can be initiated by the introduction of fuels, enabling the direct conversion of chemical energy from these fuels into mechanical energy for actuation. This section will explore the actuation mechanisms of CPs.

**Fig. 3:**
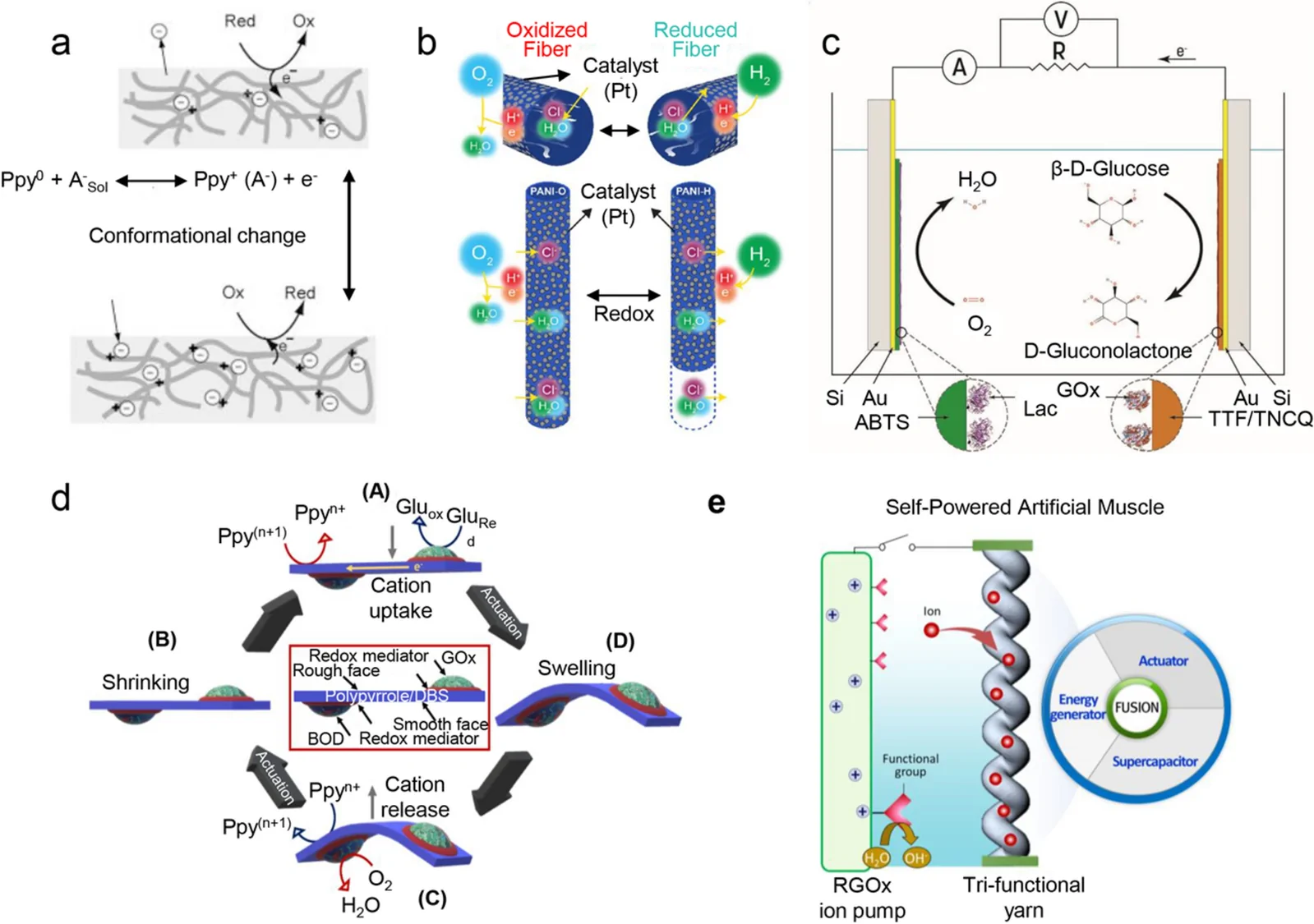
Polymer actuators driven by fuel-induced structure change. **a** Schematic illustration of anion-exchanging PPy actuator driven by the redox reaction-induced conformational change [65]. **b** Schematic illustration of the working mechanism for a reversible fuel-powered PANI fiber actuator [45]. **c** Working principle of a trilayer glucose-powered artificial muscle [66]. **d** Schematic illustration of the bio-electrochemical actuator and its actuation mechanism [71]. **e** Schematic diagram of self-powered tri-functional coiled CNT yarn muscle [131]

The most prominent CPs utilized in soft actuators include polyaniline (PANI) [32, 45], poly(3,4-ethylenedioxythiophene) (PEDOT) [61, 62], and particularly polypyrrole (PPy) [63, 64]. In 2009, Küttel et al. used PPy to generate a reversible stroke through mechanisms of redox-induced ion intercalation and deintercalation. This process involves conformational changes in the molecular chains, leading to macroscopic deformation (Fig. 3a) [65]. During actuation, the influx of solvated ions causes the polymer to expand, while the outward movement of ions results in contraction. Nonetheless, their actuation typically requires a liquid electrolyte medium.

To address the challenges associated with liquid electrolyte environments, recent research has led to the creation of solid-state actuation architectures. For example, Naraghi et al. demonstrated a fully solid-state hydrogen–oxygen system that utilizes wet-spun polyaniline (PANI) fibers combined with platinum catalysts. In this setup, alternating flows of H_2_ and O_2_ gas facilitate redox cycles through dynamic intercalation and deintercalation of Cl^−^ ions (Fig. 3b) [45]. Consequently, this actuator can reversibly contract by 3.8% while achieving an impressive energy density of 120 J kg^−1^. This fuel-powered actuator operates without the need for electrodes or an electrolyte medium, thereby providing safer and more practical applications while maintaining high energy conversion efficiency.

More recent advancements have focused on integrating biofuels with conductive polymer actuators [66–68]. One particularly innovative approach involves using enzymes to generate the necessary electrical charge, allowing for the direct extraction of fuel components from surrounding fluids. For example, Mazar et al. designed a multilayer heterostructure actuator that consists of PPy, gold, and polyvinylidene fluoride (PVDF) and uses biofuel glucose in the presence of oxygen (Fig. 3c) [66]. This actuator incorporates glucose oxidase (GOx) and laccase enzymes within its bioelectrodes, allowing them to convert glucose and oxygen into electricity. The generated electricity activates the electroactive polymer, causing it to bend and enabling autonomous power generation in physiological fluids. However, the reactions involving laccase and glucose oxidase may lead to asymmetric electron transfer, potentially resulting in asymmetric movements. To achieve balanced charge transfer, it is essential to optimize the ratio of laccase to glucose oxidase enzymes and/or adjust the mediators that facilitate charge transfer from the enzymes to the conductive polymer layer [69, 70].

While recent advancements are promising, creating a fully autonomous artificial muscle that can execute reversible motion loops powered by biochemical reactions remains a significant challenge. Notably, Arnaboldi et al. have made substantial progress by creating spatially graded enzyme interfaces within PPy actuators (Fig. 3d) [71]. The structural asymmetry of these actuators significantly enhances their dynamic feedback mechanisms. When immersed in glucose solutions, these devices exhibit autonomous oscillatory motion at 5 Hz, driven by the combined effects of anode-induced contraction and cathode recovery through an oxygen gradient. This operation results in centimeter-scale peristaltic movement, effectively mimicking biological functions in physiological environments. The researcher successfully managed the oxygen fuel by manipulating the bending behavior of PPy, which allowed precise regulation of fuel content during operation, thereby facilitating reciprocating motion. Such biomimetic locomotion strategies, using enzymatic biofuel, offer innovative solutions for medical robotics applications, including targeted drug delivery and minimally invasive surgical systems.

### Fuel-Powered Muscles Using the Generated Electricity From Fuels

Fuel-powered artificial muscles excel at converting the chemical energy of fuels into mechanical energy. However, for advanced artificial muscles to be truly effective, they must mimic the systemic, structural, and actuation properties of natural muscles. Sim et al. present a self-powered artificial muscle (Fig. 3e) that closely resembles myofibrils in these key characteristics [53]. This muscle operates through a two-step energy conversion process, first transforming chemical energy from fuel into electrical energy, and then into mechanical energy. It uses coiled multi-walled carbon nanotube (MWCNT) yarns, which serve three essential roles: function as a generator, a supercapacitor, and an actuator. This innovative yarn can generate an open-circuit voltage of 360 mV and a power density of 0.23 mW m^−2^. The produced electricity allows the yarn to contract autonomously due to ion-induced volume expansion, achieving a tensile stroke of 0.14% and a contractile work capacity of 279 kJ m^−2^. This technology greatly exceeds previous artificial muscles, achieving a fourfold increase in tensile stroke compared to fuel cell-powered alternatives [37]. Furthermore, it boasts an astonishing contraction speed that is 4700 times faster (2.3 s) than that of biofuel cell actuators, attributed to the rapid energy discharge capabilities of the supercapacitor. Remarkably, this type of fuel-powered muscle serves dual functions as both a fuel cell electrode and a supercapacitor electrode [60]. By combining energy conversion and actuation into a single compact platform, this innovation effectively addresses the challenges of miniaturization and system complexity. However, the complexities associated with controlling the reaction process are significant.

In summary, the actuation performance of fuel cell muscles is shown in Table 1. Although they demonstrate high performance, these muscles still face specific difficulties. One notable issue is that the incorporation of catalysts into the yarn muscles may reduce the yarn’s capacitance, leading to a limited actuation stroke. Additionally, the actuation speed is quite slow due to the inactivity of the catalyst. And these muscles must operate in an electrolyte, which poses challenges for packaging. Further improvement on both two-compartment and one-compartment fuel-powered actuators could focus on the development of highly efficient, nanoscale catalysts, which could enhance reaction rates and increase actuation speeds.

**Table 1:** Performances of fuel-powered actuators using charge transfer-induced actuation

	Material (Name@catalyst)	Fuel system	Max deformation	Work/Power density	Response time	Refs.
Ion-transfer	CNT@Pt	H_2_	~ 0.035% strain		30 min	[54]
	CNT@Fe₃[Co(CN)_6_]_2_	H_2_O_2_	4% stroke	0.123 J g^−1^ @35 MPa	20 s	[58]
	MWNT@SEBS	H_2_O	0.14% stroke	279 kJ m^−3^	2.3 s	[131]
	MWNT@Pt	Zinc	5.17% stroke	0.6 J g^−1^	25 s	[132]
	PI/Au/PPy	Fe_4_[Fe(CN)_6_]_3_ /C_6_H_8_O_6_	0.4%		80 ~ 280 s	[65]
	PPy/DBS@GOx/BOD	C_6_H_12_O_6_, O_2_	1 mm s^−1^ (0.5 cm × 1.5 cm)		0.15 ~ 1.1 s	[71]
	PANI@Pt/C:P407	H_2_, O_2_	~ 3.8% @0.75 MPa	120 J kg^−1^	50 s	[45]
	PPy/Au/PVDF/Au/PPy@ABTS/TTF/TNCQ	C_6_H_12_O_6_,O _2_	Bending 3 mm (0.5 cm × 2 cm)	0.27 μW cm^−2^	3 h	[66]

## Fuel-Powered Thermal Actuators

In general, the reaction between fuel and a catalyst involves simultaneous electron transfer, which is invariably accompanied by the generation of heat. In addition to the actuation module caused by ion transfer and structure change associated with the fuel reaction, the generated heat can also be utilized to activate smart materials such as shape memory alloys (SMAs) [72] and liquid crystal elastomers (LCEs) [73–75]. Actuators that harness heat generated from fuel reactions are referred to as fuel-powered thermally actuators. These actuators convert the chemical energy of fuel into thermal energy, which is subsequently converted into mechanical energy.

### Fuel-Powered Thermal SMA Actuators

Shape memory alloys are favored for their unique shape memory effect, which involves intrinsic temperature-sensitive austenite–martensite phase transformations, as well as high power densities (~ 50 kW kg^−1^) [76]. At elevated temperatures, the alloy exists in the austenite phase. When the temperature falls below the critical transformation point, the alloy structure transforms into the martensite phase, resulting in dimensional deformation. When the deformed martensite is reheated to the reverse phase transformation temperature, it reverts to its original austenite phase. However, the high temperature required to activate SMAs is typically achieved through direct electrical heating or external heat sources, necessitating the use of supplementary devices and introducing energy inefficiencies. Research has shown that fuel combustion produces large thermal energy, which can be utilized to generate the heat necessary to activate SMAs [72].

**Fig. 4:**
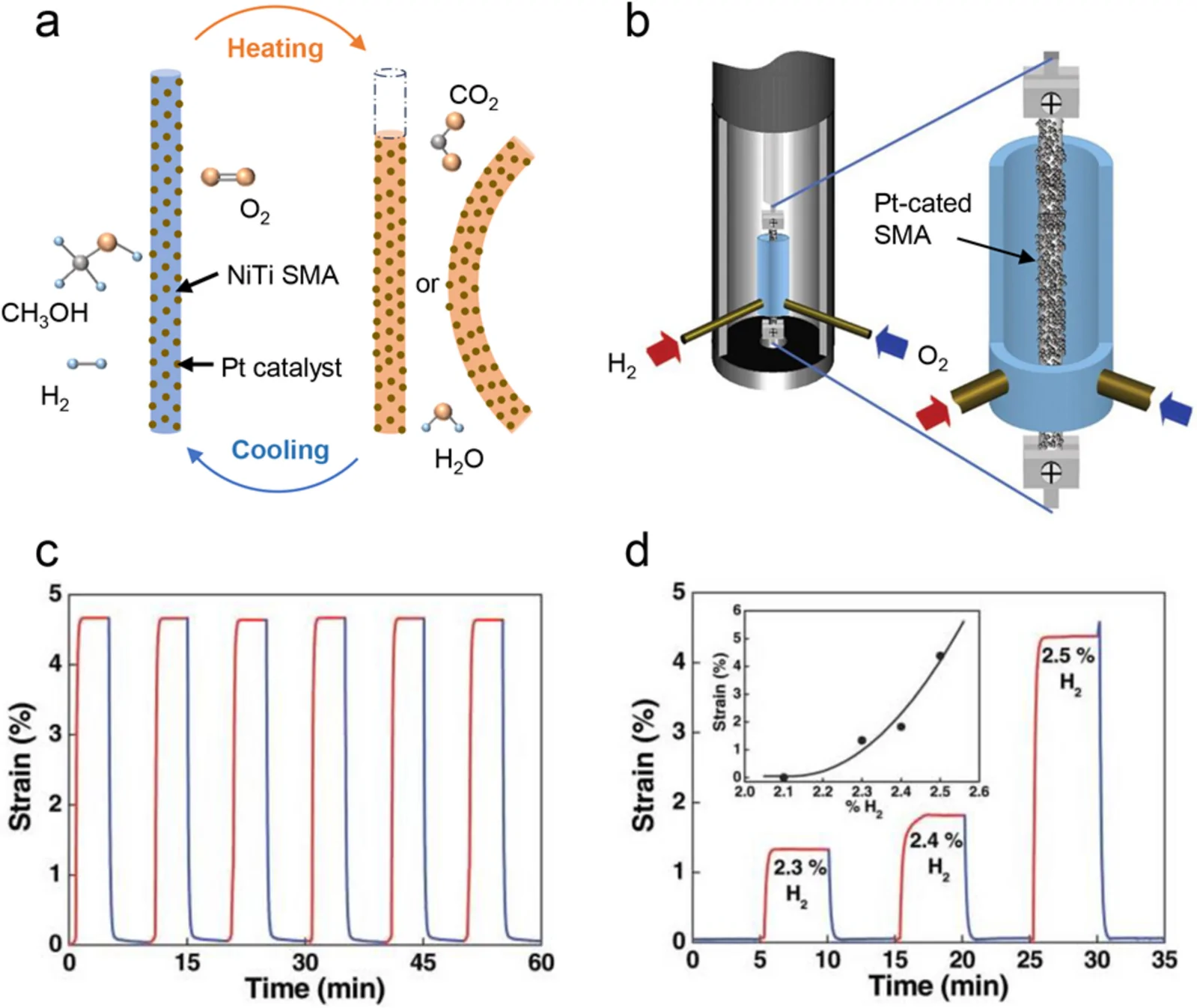
Fuel-induced thermally driven NiTi wire actuator. **a** Actuation mechanism for a Pt-coated NiTi composite wire. **b** Schematic illustration of the fuel-powered NiTi artificial muscle mounted in the dynamic mechanical analyzer used for measurements [54]. **c** Actuator strain versus time during exposure of the chemically powered actuator to a mixture of N_2_, hydrogen (2.5%), and oxygen (50%)(red curves) and during exposure to pure oxygen (blue curves) [54]. **d** The dependence of actuator strain on the H_2_ volume % in the fuel [54]

Baughman’s group first introduced a fuel-powered muscle using a nickel-titanium (NiTi) alloy wire coated with Pt catalyst [54]. The muscle operates by converting the chemical energy of fuels like hydrogen, methanol, or formic acid into thermal energy (Fig. 4a). When the fuel interacts with an oxidant (e.g., oxygen or air) and the catalyst-loaded NiTi wire, a chemical reaction takes place that generates heat. This heat elevates the wire’s temperature beyond its austenite phase-transition threshold, resulting in contraction and mechanical work, which can be measured using a dynamic mechanical analyzer (DMA), as illustrated in Fig. 4b. Once the fuel supply is interrupted, the reaction ceases, allowing the wire to cool below the martensitic-phase-transition temperature, which enables it to gradually revert to its original length (Fig. 4c, d). This fuel-oxidant powered muscle achieves a reversible contraction of 5% under a stress of 150 MPa, showcasing a stress generation capability that is 500 times greater than that of human skeletal muscle [77].

Drawing inspiration from the pioneering work of the Baughman group, Tadesse et al. developed a robotic jellyfish that utilized chemical fuel-powered SMA actuators [78]. The actuators were fabricated by coating SMA with platinum nanoparticles, which were encapsulated within CNT sheets. The used CNT sheets facilitate efficient heat transfer between the catalyst and the SMA, activating the SMA more effectively. An exothermic reaction occurs when hydrogen and oxygen gases react in the presence of a platinum catalyst, resulting in the generation of heat. Consequently, the actuator operates most efficiently at 0.1 Hz, allowing the fuel-powered bell to deform by 13.5%. This performance is comparable to that of electrically powered and naturally jellyfish [79, 80]. Overall, this robotic jellyfish harnesses a renewable hydrogen–oxygen fuel source, producing water vapor as a by-product, which not only makes it environmentally friendly but also allows for fuel regeneration.

### Autonomous, Microrobots Using Catalytic SMA Artificial Muscles

**Fig. 5:**
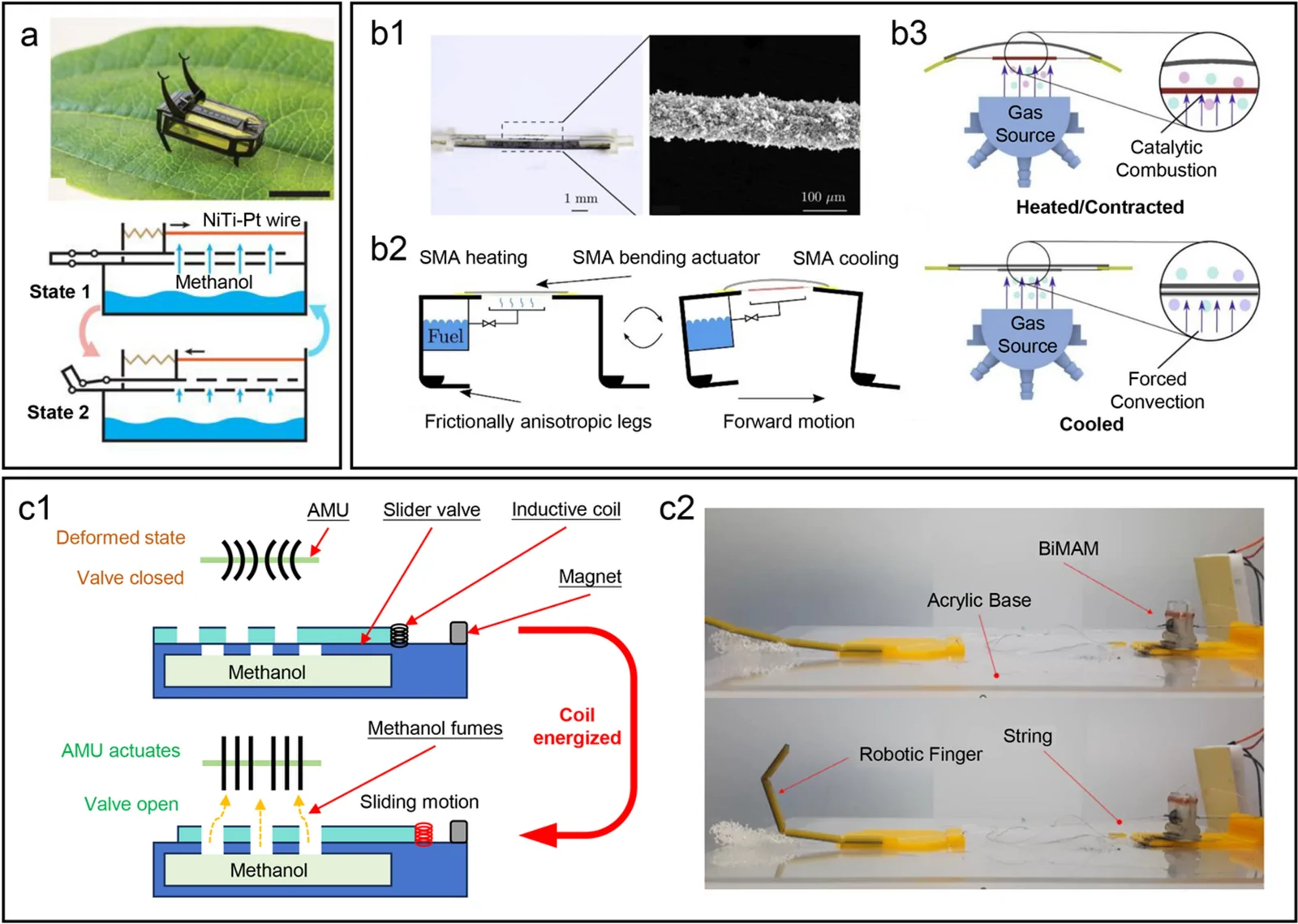
Applications of artificial muscles driven by fuel thermomechanical.** a** Schematic illustration of the robotic design of an 88-mg insect-scale autonomous robot powered by fuel and its actuation mechanism [81]. **b** Schematic illustration of a miniature catalytic combustion engine (1) Close-up picture and SEM image, (2,3) Conceptual design of a robot and miniature catalytic combustion engine [84]. **c** 1) Working principle of electronic control of actuating multi-wired AMU with chemical fuel (methanol), (2) Experimental setup of the robotic finger curling using BiMAM [85]

In addition to traditional-sized robots, such as jellyfish, fuel-powered thermal actuators show promising potential for microrobots. Many sub-gram mobile robots designed for continuous operation rely on external power sources linked by cables or electromagnetic fields, which restricts their functionality in confined environments. Fuel-powered actuators offer a groundbreaking solution for powering autonomous microrobots. An example is the RoBeetle, an 88-mg crawling robot created by Yang et al., which uses a methanol-powered SMA wire (Fig. 5a) [81]. The RoBeetle is equipped with a fuel tank that stores liquid methanol, a platinum catalyst-coated NiTi wire, and a movable plastic foreleg. When methanol undergoes catalytic combustion, it activates the SMA wire, causing it to contract and subsequently rotate the foreleg. As the SMA wire contracts, it manipulates a shutter that regulates the microvalves in charge of controlling the fuel supply. This reduction in fuel delivery slows the catalytic reaction, leading to decreased heat production and allowing the SMA wire to cool. Upon cooling, the wire gradually returns to its original length. This cycle of contraction and elongation in the SMA wire establishes a self-regulating feedback loop that governs fuel delivery and reaction rates. By maintaining this balance, the system can function autonomously at a frequency of 1–2 Hz. Consequently, the robot can achieve speeds ranging from 0.37 to 0.76 mm s^−1^ on flat surfaces as well as manage inclines of up to 10° while carrying loads of up to 230 mg (2.61 times its weight).

Note that early microrobots utilizing heat from fuel combustion were constrained to slow operation speeds under 1 Hz, primarily due to slow cyclic phase transformations [54, 78, 81, 82]. In contrast, the lithium battery-powered NiTi-based robot, known as SMALLBug [83], operates at significantly higher frequencies, around 20 Hz. This enhanced performance is achieved using extremely thin wires that facilitate rapid heating and cooling cycles, complemented by a conductive carbon fiber (CF) sheet that provides the necessary stress to activate the SME of SMA. Inspired by the fast-actuating capabilities of SMALLBug and the catalytic combustion-powered RoBeetle, Maimani et al. developed a compact catalytic combustion engine weighing just 7 mg that operates on fuel combustion (Fig. 5b1) [84]. This engine employs a 38-μm-diameter NiTi wire coated with a platinum catalyst, arranged in a looped composite structure with a 13 mm carbon fiber leaf spring, enabling periodic actuation. While hydrogen fuel contacts and ignites on the SMA surface, the engine efficiently converts chemical energy into mechanical work through the thermal cycling of the SMA wire. Remarkably, this small engine achieves an impressive power density of 39.5 μW mg^−1^, operating efficiently at a high frequency of 6 Hz. This miniaturized catalytic combustion engine serves as the basis for potential designs for a robot and engine, as illustrated in Fig. 5b2, b3.

Despite the progress in the catalytic combustion-based actuators, these actuators rely on a passive control system that employs a mechanical mechanism (spring) devoid of electronic components for catalytic heating. To improve the active control of catalytic heating in shape memory alloys, inspired by human skeletal muscles, Nagwade et al. developed a biomimetic artificial muscle (BiMAM, Fig. 5c1) that is powered by fuel and can be electronically controlled [85]. This muscle employs carbon nanotube/platinum black (CNT/Pt) nanocomposite-coated SMA wire as a bending actuator, allowing for rapid actuation through controlled catalytic heating of methanol fuel managed by an electromechanical valve system. This approach can achieve a higher energy efficiency, which is about three times higher compared to traditional Joule heat-driven SMA actuators [86, 87]. The BiMAM can contract in just 0.5 s and cools down within 4.5 s, exhibiting stable performance over 100 cycles at 0.18 Hz. Moreover, the integration of EMG control and robotic finger actuation (Fig. 5c2) underscores the potential for developing chemically powered devices for human applications.

Overall, fuel-powered SMA actuators present significant advantages by leveraging the properties of catalytic combustion and SMA phase transformation (Table 2). By using high-energy density fuels like hydrogen and methanol, these actuators overcome the energy limitations commonly faced by conventional batteries, allowing milligram-scale systems to operate over prolonged durations. Looking to the future, development efforts may focus on several key areas: First, optimizing the structural and enhancing the thermal conductivity of SMA could lead to fast response speeds. Second, integrating multimodal control systems might facilitate closed-loop feedback, opening avenues for applications in medical interventions and search-and-rescue missions. Lastly, exploring easily storable fuels and advancing miniaturized fuel storage technologies will be crucial for addressing important safety considerations.

**Table 2:** Performances of fuel-powered thermal actuators

	Material (Name@catalyst)	Fuel system	Max deformation	Work/Power density	Operating frequency (Max, Hz)	Refs.
Fuel-powered thermal actuators	NiTi@Pt	H_2_, CH_3_OH, or HCOOH	~ 8% @98 MPa	5300 kJ m^−3^, 68 W/kg (CH_3_OH), 6800 kJ m^−3^ (CH_3_OH, HCOOH)	~ 0.001	[54]
	NiTi@Pt/MWCNT	H_2_, O_2_	13.5%		~ 0.3	[78]
	NiTi@Pt	CH_3_OH	3%	96 kJ kg^−1^	~ 1	[81]
	NiTi@Pt/CF	H_2_	6.92%	5.65 W kg^−1^	6	[84]
	NiTi@Pt/CNT	CH_3_OH	25% linear stroke	226 mW@0.18 Hz	0.18	[85]

## Fuel-Powered Pneumatic Actuators

The by-products of fuel reactions are typically gaseous, which can also be harnessed for propulsion. Pneumatically powered soft robots use pressurized gas within their microchannel networks to generate motion [88–95]. Although these systems are relatively easy to manufacture and economically viable, their deployment in minimally invasive environments is hindered by the requirement of tethered components, such as pumps or tubing. Onboard gas sources are essential for powering minimized pneumatic actuators. Alternative methods, including explosive combustion [30, 96, 97] and hydrogen peroxide-based pressure source [98], offer effective solutions for generating pressure onboard. This innovation eliminates the reliance on tethered pumps and tube accessories in pneumatic actuators. This section will explore the structural design, fundamental principles, control strategies of chemical reactions, and the robotic applications of fuel-powered pneumatic actuators.

### Thermal Pneumatic Actuators Powered by Chemical Reaction of Fuel

The combustion of fuel involves a rapid and intense chemical reaction that generates a large amount of thermal energy in a brief time [99]. This thermal energy can elevate the temperature within the fuel system. According to the ideal gas law, expressed as PV = nRT, an increase in temperature leads to a corresponding increase in pressure when the fuel is confined within sealed chambers. Pneumatic actuators that utilize this thermal energy are referred to as thermal pneumatic actuators.

Methane has an energy density of around 55 MJ kg^−1^, enabling it to generate a substantial amount of energy in a short time. This characteristic enables the rapid inflation of pneumatically actuated soft robots without the need for bulky electronic pumps. A decade ago, Shepherd et al. employed methane combustion to power soft robotic actuators [100]. By enhancing spark timing and optimizing the robot’s design, they were able to achieve higher jump heights and increased energy conversion efficiency. However, controlling the direction of the jump remains a challenge.

**Fig. 6:**
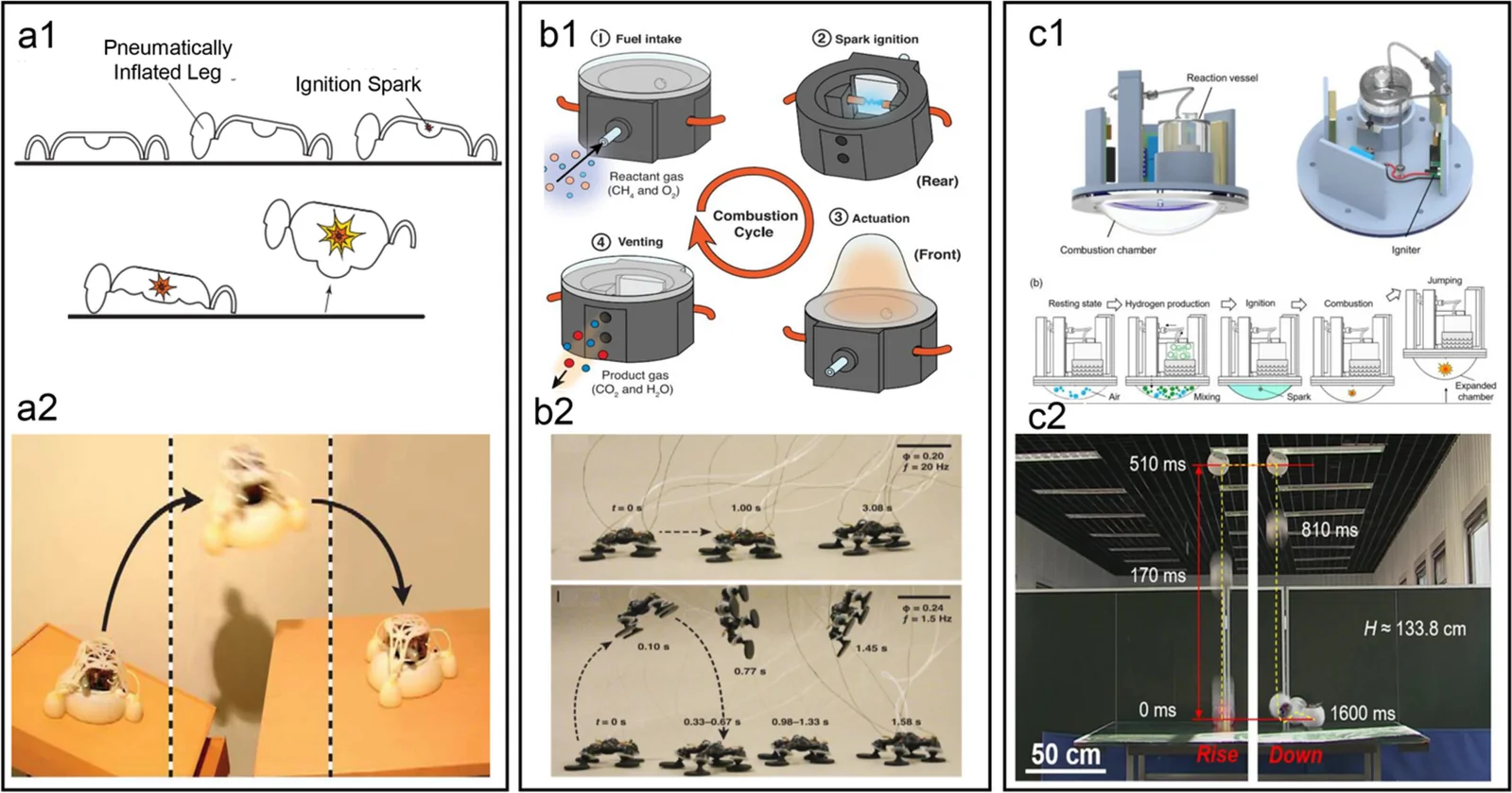
Thermal pneumatic actuators powered by the chemical reaction of fuel. **a** (1) A jumping robot design and principle of operation, (2) A jumping robot performs a targeted jump off of an angled surface onto a table [30]. **b** (1) Operation and characterization of the combustion-driven microactuator, (2) Time-lapse image of the robot performing a crawling gait and a hopping gait [31]. **c** Illustration of soft jumping robotic module based on LM-enabled hydrogen energy supply and explosion-actuated motion. (1) The structure of a soft jumping module, (2) The performance of vertical jumping [105]

To enable the robots to leap over obstacles, Bartlett et al. developed a jumping robot that integrates rigid silicon carbide ceramic combustion chambers with flexible silicone elastomer casings through 3D printing, thereby eliminating the need for complex molding techniques or assembly processes [30]. This robot primarily consists of two nested hemispherical components (Fig. 6a1). The upper hemisphere features a modulus that can vary over three orders of magnitude, ranging from roughly 1 MPa to 1 GPa. The lower hemisphere includes a small depression that stores a mixture of oxygen and butane. Igniting this gas mixture prompts the robot to jump. Surrounding the central actuator are pneumatic legs arranged in a nested hemi-ellipsoid configuration, which allows the robot to tilt its body before taking off, providing effective control over its direction of movement. This separation of power and control actuators simplifies the actuation process and enhances directional capabilities. As a result, the robot can jump to a height of 0.76 m (6 body heights) and travel laterally up to 0.15 m (0.5 body lengths) with each leap (Fig. 6a2).

When deploying the soft jumping robot for specialized applications, such as search-and-rescue missions, it is essential to prioritize both directional control and a compact structure design. Cameron et al. developed a soft combustion actuator that employs 3D-printed flame-retardant chambers and elastomeric membranes to convert the energy generated from methane-oxygen combustion into mechanical work (Fig. 6b1) [31]. This actuator achieves a linear strain of 140% at frequencies over 100 Hz and produces an impulse force greater than 9.5 N, significantly surpassing the performance of traditional dielectric elastomer and piezoelectric microactuators [101–104]. Moreover, the actuator incorporates passive flame quenching through sub-milliliter chamber geometry, allowing for cyclic operation with a peak power density of 277.2 kW kg^−1^. When assembled into a quadrupedal microrobot weighing 1.6 g, it supports multimodal locomotion, achieving vertical jumps of 59 cm and horizontal leaps of 16 cm (Fig. 6b2). Additionally, this microrobot provides a payload capacity of 22 times its body weight and enables programmable gait transitions ranging from 0 to 30 Hz, highlighting the potential for dynamic insect-scale robotics in constrained environments.

Notably, the combustion of fuel to generate gas pressure is inherently an irreversible process, which poses challenges for maintaining controllable and safe pressure regulation. One effective strategy to mitigate this issue is the use of catalysts to manage fuel reaction precisely. Liu et al. developed a soft jumping robot that is equipped with an independent hydrogen supply, where the reaction is catalyzed by liquid metal (LM) and fueled by the combustion of the resulting hydrogen (Fig. 6c1, c2) [105]. This innovative design generates hydrogen gas in real-time by employing gallium-based liquid metal to facilitate the reaction between aluminum and water. The robot seamlessly integrates the fuel supply, combustion, and control systems, providing a coherent operational framework.

### Pneumatic Actuators Powered by the Chemical Reaction of Fuel, Operating Without Heat

Pneumatic actuators powered by fuel combustion deliver high energy density and quick response times. These actuators operate by igniting a mixture of fuel and oxidant using an electric spark, resulting in the rapid creation of high-pressure gases, typically around 500 kPa [30, 31, 105]. However, this combustion process can lead to thermal energy loss and the emission of hazardous by-products, including carbon monoxide (CO) and nitrogen oxides (NO). Additionally, achieving precise control over the combustion rate presents considerable challenges [106]. To address these issues, extensive research has been directed toward methods for generating gas from fuel without generating heat. Approaches such as microfluidic control of pressurized gas and reversible management of chemical reactions through thermoelectric devices are being investigated.

**Fig. 7:**
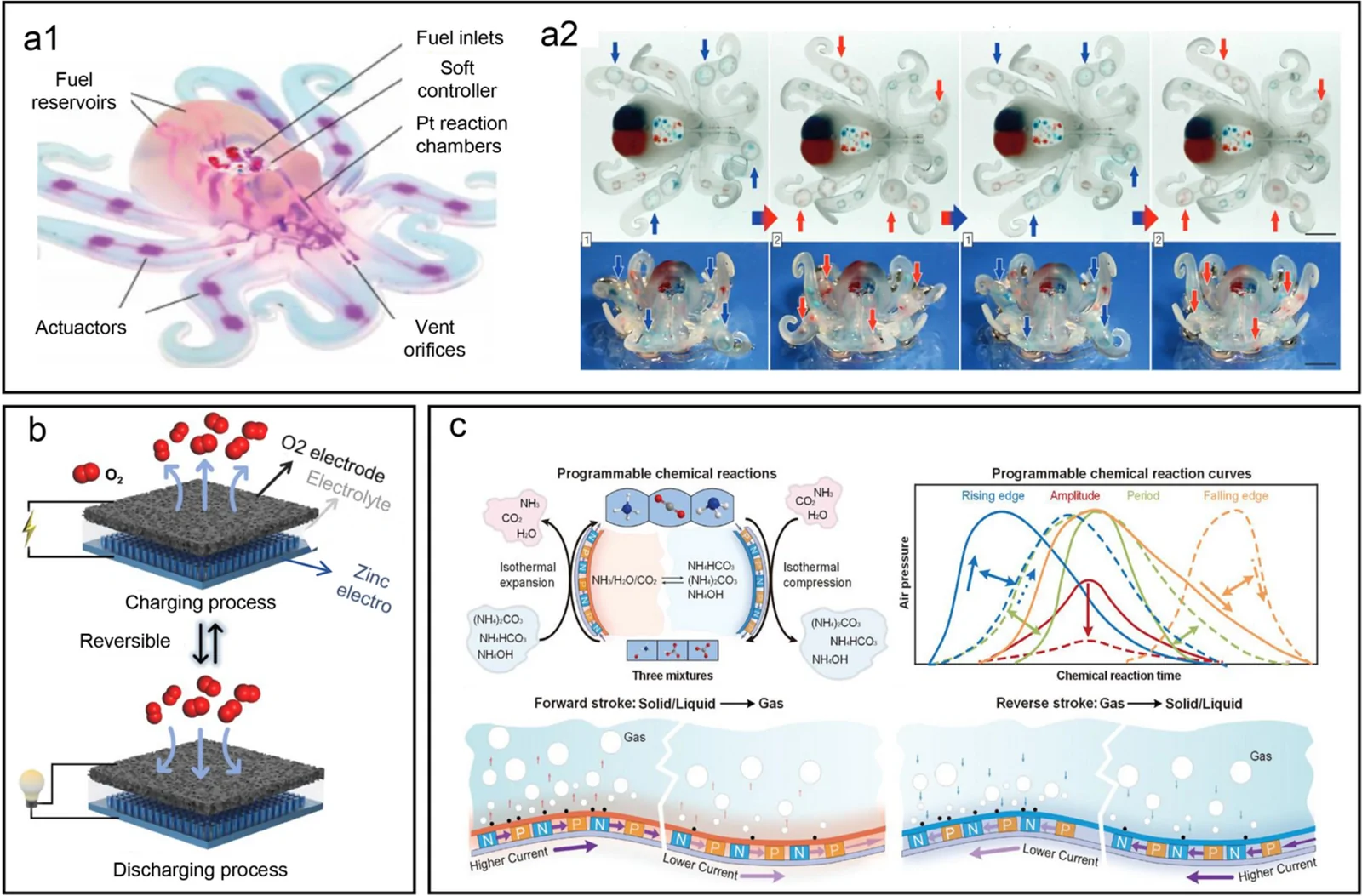
Pneumatic actuators powered by the chemical reaction of fuel without producing heat.** a** (1) Schematic illustration of a fully soft, autonomous robot that is controlled via the embedded microfluidic soft controller and powered by monopropellant decomposition, (2) The Octobot autonomously alternating between blue and red actuation states [98]. **b** Schematic illustration of EPB (electrochemical pneumatic battery) charging and discharging process [111]. **c** Concept and working principle of programmable chemical reactions [113]

The decomposition of chemical fuel into gas generally occurs through a mild reaction [106–110]. For example, Wehner et al. utilized the decomposition of onboard hydrogen peroxide to generate gas pressure for powering an entirely soft, nontethered robot, named Octobot [98]. This robot utilizes microfluidic logic to autonomously control fluid flow, which drives the catalytic decomposition of a hydrogen peroxide fuel source (Fig. 7a1). The gas generated during this reaction inflates the downstream fluidic networks, enabling actuation (Fig. 7a2). However, this process yields relatively low pressure, around 50 kPa [98], and is irreversible.

Electrochemical reactions offer an effective solution to the challenges mentioned above and can be used to regulate the gas generation rate. Ge et al. designed a reversible electrochemical pneumatic battery (EPB) that utilizes a zinc-air battery to supply both electrical and pneumatic power [111]. The battery operates by releasing or absorbing O_2_ during the charging or discharging process, respectively (Fig. 7b). By utilizing oxygen from a metal-air battery as the exclusive gas source, the EPB achieves an extensive pressure range from − 1 to 7 bar. This process is quiet, controllable, and inherently reversible. The EPB shows great potential for self-powered robotic applications, including layer jamming variable-stiffness plate, negative pressure-driven origami, and positive pressure-driven bellow actuators.

Thermoelectric materials enable temperature regulation through the Peltier effect, making them valuable for manipulating the equilibrium state of chemical reactions [112]. Their superior heat management capabilities allow for programmable chemical processes and controlled reactions. For example, Qu et al. introduced a programmable chemical reaction system designed to power soft pneumatic actuators [113, 114]. They used thermoelectric materials to modify reaction conditions, shift reaction equilibrium, and adjust reaction rate (Fig. 7c). This innovative system can generate remarkable pressures of nearly 6 MPa and exert forces of approximately 18 kN. Moreover, it can power artificial muscles that produce an output force of 0.4 kN under static conditions and achieve a stroke of 25% when lifting a 10 kg load. With a lightweight design of less than 500 g and biocompatible reactants (ammonia and ammonium), this system is well-suited for seamless integration into assistive wearables, micro-tactile interfaces, and medical robots.

### Microrobots Propelled by Bubbles Produced from Fuel Chemical Reactions

Conventional nanoshuttles used for drug delivery mainly rely on Brownian motion, which is inefficient and slow [101, 102]. There is a strong need to develop autonomous drug delivery robots capable of quickly and accurately encapsulating, transporting, and releasing substances [103]. The gas generated from fuel chemical reaction can be used for drug delivery vehicles or nanoshuttles as well, aiding in the transport of therapeutic and diagnostic agents. Fuel-powered nanoshuttles enhance drug delivery by catalyzing fuel decomposition on functionalized substrates. Because of their excellent biocompatibility, this technology plays an important role in implantable medical devices.

**Fig. 8:**
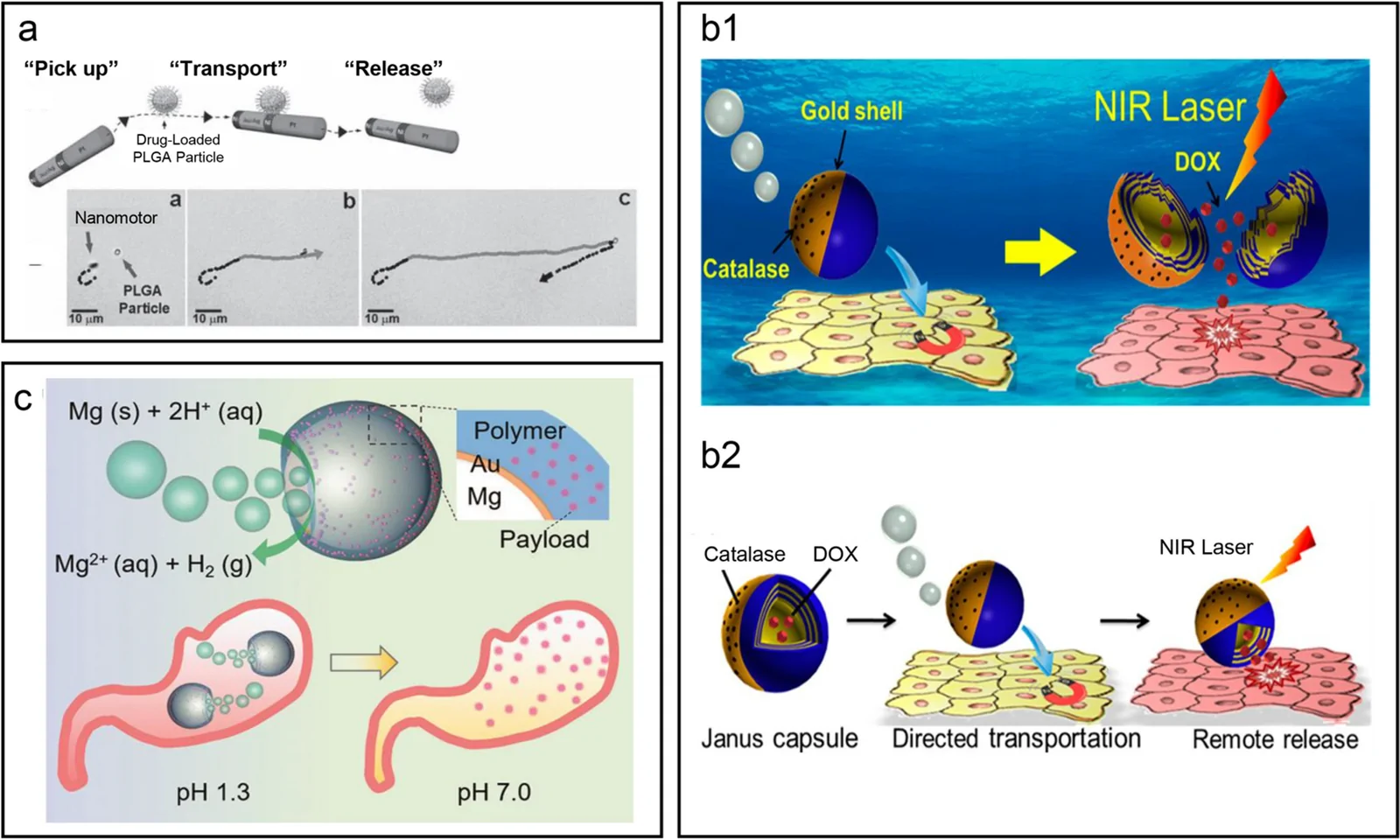
Microrobots propelled by bubbles generated from the chemical reaction of fuel. **a** Scheme depicting the dynamic pickup, transport, and release of drug-loaded PLGA particles using a nanoshuttle [115]. **b** (1) Scheme of the targeted movement and light-triggered drug release of Janus capsule motors, (2) Scheme of the targeted movement and light-triggered drug release of Janus capsule motors [119]. **c** Illustrations of an acid-powered Mg-based micromotor and its acid neutralization mechanism [127]

Some nanoshuttles operate based on the decomposition of hydrogen dioxide (H_2_O_2_). Kagan et al. first reported a fuel-powered nanoshuttle that could pick up, transport, and release common drug carriers, including biocompatible and biodegradable polymeric particles and liposomes [115]. This nanoshuttle utilized a 5 wt% H_2_O_2_ solution as its fuel source and Pt as the catalyst (Fig. 8a). The decomposition of H_2_O_2_ on the nanoshuttle’s surface generates oxygen bubbles, and the force produced during the formation and release of these bubbles propels the nanoshuttle, allowing it to efficiently transport drug-loaded particles like PLGAs and liposomes. Notably, these nanoshuttles achieve transport speeds that are approximately three orders of magnitude faster than those achieved by Brownian motion [116–118].

Although the utilization of Pt as a catalyst accelerates the decomposition of H_2_O_2_ and increases the transport speed of nanoshuttles, the biotoxicity associated with H_2_O_2_ and the catalyst Pt itself can present challenges for applications in biological contexts. To mitigate the impact of H_2_O_2_ and metal catalysts on living organisms, Wu et al. developed a novel hybrid biocatalytic Janus motor, which uses hollow polyelectrolyte multilayer capsules and a catalytic enzyme (Fig. 8b1, b2) [119]. Compared to actuators using Pt catalysts, this enzyme-based system uses lower concentrations of H_2_O_2_ [120].

To further broaden the applications of fuel-powered actuators in biomedical fields, researchers are increasingly investigating alternative fuels and catalytic methods to demonstrate better biocompatibility. Pantarotto et al. illustrated that covalently attaching glucose oxidase (GOx) and catalase (CAT) to carbon nanotube (CNT) fibers enables actuation [121, 122]. When glucose reacts with the enzymes on the CNT, it successfully generates oxygen bubbles and then drives the fibers. The use of enzymes enables the programming of motion trajectories.

In contrast to nanoshuttles that passively respond to environmental cues by moving to specific locations, some advanced micromotors can manipulate their surroundings, such as regulating pH levels, to create optimal conditions for the release of their payload [123–126]. For instance, Li et al. developed a magnesium (Mg)-based micromotor coated with a pH-sensitive polymer, as shown in Fig. 8c [127]. When the micromotor interacts with gastric juice, hydrogen ions (H^+^) react with the Mg, generating hydrogen bubbles that propel the motor while simultaneously adjusting the pH of the gastric fluid. The micromotor can temporarily neutralize the acidity of gastric fluid in vitro and in vivo experiments, allowing for the precise release of therapeutic agents at optimal pH levels. Its effectiveness in treating severe gastritis was demonstrated through experiments on a mouse model [128].

For bio-robotic applications, certain catalysts utilized in fuel-powered actuators present noteworthy risks. For instance, platinum-based nanoparticles can infiltrate cells, leading to excessive production of reactive oxygen species. This can damage DNA and proteins, potentially resulting in tissue fibrosis or immune rejection [129]. Similarly, cobalt-based cyanide complexes risk releasing toxic cyanide ions that inhibit mitochondrial function and disrupt cellular energy metabolism, posing dangers specifically to nerve and cardiac cells [130]. Additionally, nickel-based catalysts can disrupt calcium signaling, possibly triggering inflammation and increasing the risk of allergic reactions. These toxic catalysts, along with metabolic disruptions caused by fuels, highlight the important safety concerns in biological applications. Future research aimed at advancing high-performance fuel-powered biorobots must prioritize enhancing safety and biocompatibility. A variety of strategies can be explored, such as developing environmentally friendly catalysts, like enzymes, to replace metal-based options. Additionally, innovative packaging methods could facilitate the integration of catalysts and fuels into encapsulated systems, thus mitigating the associated risks.

All in all, while chemical fuels offer high energy density, most systems suffer from low conversion efficiency (Table 3). Volumetric limitations in microscale systems (e.g., sub-milliliter chambers in insect-scale robots) further restrict their sustained operation, requiring frequent refueling or replacement. In addition, current systems often rely on irreversible chemical reactions (e.g., hydrogen peroxide decomposition, combustion) that lack precise control over pressure output and timing. Reversible reactions show great potential but require complex thermal management via thermoelectric materials to adjust reaction equilibria, adding system complexity. Seamlessly embedding gas chambers within soft actuators without compromising flexibility is challenging. To mitigate the impact of catalysts and fuels, advancements can be achieved through biological functional substitution, material barrier design, and system integration innovation.

**Table 3:** Performances of fuel-powered pneumatic actuators

	Material (Name@catalyst)	Fuel	Max deformation/speed	Work/Power density	Operating time/frequency	Refs.
Fuel-powered pneumatic actuators	3D printed chambers, ignition	C_4_H_10_	Jump to 0.76 m (six body heights)			[30]
	Hydrogel-based inks and PDMS	H_2_O_2_	80° (bending)	1.44 kJ g^−1^	12.5 min	[98]
	Nylon, soft rubber, and HDPE	H_2_	Jump to 133.8 cm @110 kPa		~ 300 s	[105]
	EPX-86 FR	CH_4_	7 mm (140%)	888.3 W kg^−1^ (continuous)	> 100 Hz	[31]
	Thermoelectric ceramic shelled P–N junction @Bi_2_Te_3_	NH_3_/H_2_O/CO_2_	25% stroke @10 kg			[113]
	CNT or alloy nanomotors	H_2_O_2_	16 μm s^−1^			[115]
	SiO_2_@PSS/PAH	H_2_O_2_	232 μm s^−1^ @2 wt% 4.2 μm s^−1^ @0.1 wt%			[119]
	Mg@Au/EUDRAGIT® L100-55	HCl	60 μm s^−1^		24 h	[127]

## Conclusions and Outlook

Fuel-powered actuators, which convert the chemical energy of fuel into mechanical energy, represent a revolutionary advancement in self-sustaining actuation technologies. In this review, we summarize the various working principles, typical examples, and applications of fuel-powered actuators. We also highlight the recent progress aimed at improving energy conversion efficiencies and examine the role of these actuators in autonomous robots. Unlike battery-powered actuators, fuel-powered actuators can sustain prolonged operation in compact environments. The conversion of fuel energy into mechanical work can be achieved through several methods, including charge transfer-induced ion injection or structural changes, fuel-powered thermal actuation, and fuel-powered pneumatic actuation. To fully understand the relative advantages and trade-offs of each actuation mechanism, we have outlined several key strategies, which are shown in Table 4.

**Table 4:** Fuel-powered actuators: stroke, power densities, operational frequencies, and issues

	Working principle	Actuation performance	Power density (Max.)	Operational frequency	Autonomy(A)/Biocompatibility(B)/Controllable(C)	Issues & guidelines
Fuel-electrochemical	Ion Injection	0.03 ~ 5.2% Stroke	~ 0.6 J g^−1^	0.0002 ~ 3 Hz	C	Low frequency, need electrolytes Developing solid-state electrolytes
	Structural change	0.4 ~ 4% Stroke	~ 0.0027 W m^−2^	0.0001 ~ 2.5 Hz	C	Low frequency
	Electricity	Bending/Crawling	–	Continuous	A	Fuel supply regulation
Fuel-thermal	Thermal	3% ~ 25% Stroke	0.2 ~ 68 W kg^−1^	0.001 ~ 6 Hz	A/C	Low thermal conductivity and efficiency Increasing specific surface area
Fuel-pneumatic	Combustion	Jumping (0.7 ~ 1.3 m)	~ 0.8 kW kg^−1^	0 ~ 100 Hz	C	Intense reaction, high temperature Increase controllability and sealing
	Decomposition	Bending/Propulsion	–	Continuous	A/B/C	Slow speed, low output, and toxic Develop new catalysts and fuel systems

The detailed comparisons and future directions are summarized as follows:

1. Fuel-powered artificial muscles using charge transfer-induced actuation: These actuators utilize charge transfer to adsorb solvation ions or cause structure change triggered by redox reactions, delivering high energy conversion efficiency. However, their practical implementation is constrained by inherent challenges, including a slow response speed of less than 1 Hz, attributed to ionic migration kinetics, as well as packaging issues related to the required electrolytes. Utilizing solid electrolyte can eliminate the need for liquid ones, making them suitable for flexible and wearable actuators. One possible application of fuel-powered chemical actuator is in autonomous robotic fish or space exploration, which could be powered by stored fuel or methane (CH_4_) sourced from the ocean environment. Such robots could operate for longer time, compared to battery-powered robotic fish or space vehicle.
2. Fuel-powered thermal actuators: The thermal energy generated from fuel reactions can be harnessed to power smart materials like shape memory alloys (SMA) and liquid crystal elastomers (LCE). These actuators demonstrate high actuation strokes (> 20%) and high power densities, enabling capabilities in insect-scale jumping or swimming robots. However, they still face some issues due to thermal inertia effects, resulting in response frequencies typically under 10 Hz, as well as low energy conversion efficiency, typically below 1%. Enhancing the surface structure and thermal conductivity of SMA could result in faster response speeds. Furthermore, integrating AI-integrated multimodal control systems may enable closed-loop feedback, opening avenues for applications in medical interventions and search-and-rescue missions.
3. Fuel-powered pneumatic actuators: Pneumatic-driven artificial muscles primarily operate by using pressurized gas or the propelling force of the gas. The intense combustion of fuel generates high gas pressure sharply, which can be used for powering robots. This approach faces several challenges, including maintaining a continuous fuel supply and on–off control of fuel reaction. Future research should concentrate on creating biomimetic enzyme-catalytic materials to enhance efficiency. Another approach is to use the mild reaction of gas generation from fuel (e.g., H_2_O_2_ decomposition) to power an actuator, facilitating pneumatic actuation without thermal impacts. These bubble propulsion actuators can be used to deliver drugs to target locations of the human body.

While fuel-powered actuators offer notable benefits, including high energy density and versatile functionality, the efficient transformation of chemical energy into mechanical work continues to pose significant challenges. Progress in this field necessitates optimization of fuel-to-actuation conversion efficiency alongside essential factors such as biocompatibility, prolonged actuation duration, and directional control.
